# Identification of Novel Compounds Inhibiting Chikungunya Virus-Induced Cell Death by High Throughput Screening of a Kinase Inhibitor Library

**DOI:** 10.1371/journal.pntd.0002471

**Published:** 2013-10-31

**Authors:** Deu John M. Cruz, Rafaela M. Bonotto, Rafael G. B. Gomes, Camila T. da Silva, Juliana B. Taniguchi, Joo Hwan No, Benoit Lombardot, Olivier Schwartz, Michael A. E. Hansen, Lucio H. Freitas-Junior

**Affiliations:** 1 Center for Neglected Diseases Drug Discovery (CND3), Institut Pasteur Korea, Seongnam-si, Gyeonggi-do, South Korea; 2 Universidade Feevale, Novo Hamburgo, Rio Grande do Sul, Brazil; 3 Universidade Federal de Uberlândia, Uberlândia, Minas Gerais, Brazil; 4 Universidade Estadual do Rio Grande do Sul - Campus Novo Hamburgo, Novo Hamburgo, Rio Grande do Sul, Brazil; 5 Universidade Estadual Paulista “Júlio de Mesquita Filho”-Campus Araraquara, Araraquara, São Paulo, Brazil; 6 Image Mining Group (IMG), Institut Pasteur Korea, Seongnam-si, Gyeonggi-do, South Korea; 7 Virus and Immunity Unit, Department of Virology, Institut Pasteur, Paris, France; National Institutes of Health, National Center for Advancing Translational Sciences, United States of America

## Abstract

Chikungunya virus (CHIKV) is a mosquito-borne arthrogenic alphavirus that causes acute febrile illness in humans accompanied by joint pains and in many cases, persistent arthralgia lasting weeks to years. The re-emergence of CHIKV has resulted in numerous outbreaks in the eastern hemisphere, and threatens to expand in the foreseeable future. Unfortunately, no effective treatment is currently available. The present study reports the use of resazurin in a cell-based high-throughput assay, and an image-based high-content assay to identify and characterize inhibitors of CHIKV-infection in vitro. CHIKV is a highly cytopathic virus that rapidly kills infected cells. Thus, cell viability of HuH-7 cells infected with CHIKV in the presence of compounds was determined by measuring metabolic reduction of resazurin to identify inhibitors of CHIKV-associated cell death. A kinase inhibitor library of 4,000 compounds was screened against CHIKV infection of HuH-7 cells using the resazurin reduction assay, and the cell toxicity was also measured in non-infected cells. Seventy-two compounds showing ≥50% inhibition property against CHIKV at 10 µM were selected as primary hits. Four compounds having a benzofuran core scaffold (CND0335, CND0364, CND0366 and CND0415), one pyrrolopyridine (CND0545) and one thiazol-carboxamide (CND3514) inhibited CHIKV-associated cell death in a dose-dependent manner, with EC_50_ values between 2.2 µM and 7.1 µM. Based on image analysis, these 6 hit compounds did not inhibit CHIKV replication in the host cell. However, CHIKV-infected cells manifested less prominent apoptotic blebs typical of CHIKV cytopathic effect compared with the control infection. Moreover, treatment with these compounds reduced viral titers in the medium of CHIKV-infected cells by up to 100-fold. In conclusion, this cell-based high-throughput screening assay using resazurin, combined with the image-based high content assay approach identified compounds against CHIKV having a novel antiviral activity - inhibition of virus-induced CPE - likely by targeting kinases involved in apoptosis.

## Author Summary

Recent outbreaks and expanding global distribution of Chikungunya virus (CHIKV) in different regions of Asia, Africa and Europe necessitates the development of effective therapeutic interventions. At present, only two antiviral compounds (chloroquine and ribavirin) that inhibit viral infection *in vitro* have been used in clinical cases of chikungunya infections. However, neither of these compounds have shown strong efficacy in vivo. Recent attempts to identify new antiviral candidates for CHIKV using cell-based phenotypic approach have been reported. In this study, we developed a simple cell-based high-throughput assay using resazurin to identify potential anti-CHIKV compounds. This high-throughput assay is based on the metabolic reduction of resazurin to the highly fluorescent resorufin by viable cells as an indicator of activity against CHIKV-induced CPE. We screened 4,000 small molecules belonging to the BioFocus kinase inhibitor chemical library and found a cluster of related molecules with antiviral activity against CHIKV. Finally, we characterized the putative mode of action of these active compounds using an image-based high content assay and conventional virological methods (*i.e.*, virus yield reduction assay, microneutralization assay).

## Introduction

Chikungunya virus (CHIKV) is a mosquito-borne pathogen belonging to the Semliki Forest antigenic complex of the genus *Alphavirus*, family *Togaviridae* [1]. CHIKV has a single, positive strand linear RNA genome of approximately 11.7 kb and is comprised of two open reading frames (ORF): a 5′ non-structural ORF encoding the non-structural polyprotein, and a 3′ structural ORF that encodes the structural polyprotein of the virus [2]. A unique feature shared by CHIKV with other members of the family *Togaviridae* is the translation of the structural polyprotein from the 26S mRNA, which is internally transcribed from the negative strand template through the initiation of the 26S subgenomic promoter, located at the junction region between the non-structural and structural ORFs. Based on the genomic organization of other related alphaviruses, the CHIKV genome is considered to be: 5′-nsP1-nsP2-nsP3-nsP4-junction region-C-E3-E2-6k-E1-poly(A)-3′ [3]. CHIKV virions have a spherical capsid with icosahedral symmetry surrounded by a lipid bilayer envelope (about 70 nm in diameter) derived from the host cell membrane during virus budding. Two viral glycoproteins embedded in the envelope, E2 and E1, direct the attachment to the host cell membrane and subsequent fusion with the endosomal membrane, respectively [4], [5]. CHIKV is transmitted between human hosts by blood-feeding female mosquitoes of the *Aedes* species, particularly *Ae. aegypti* and *Ae. albopictus*, often resulting in a clinical condition known as chikungunya fever (CHIKF) [6], [7]. Clinical symptoms of CHIKV infection are similar to that of other arthrogenic alphaviruses like Sindbis virus (SINV), Mayaro virus (MAYV) O'nyong-nyong virus (ONNV) and Ross River virus (RRV), with arthralgia being the hallmark feature [8].

CHIKV was first isolated in Tanganyika (now called Tanzania) in 1953 [9], and has become endemic in Africa, India and Southeast Asia. Several imported cases of CHIKF have also been reported in Europe [10]. The occurrence of chikungunya epidemics has been unpredictable, with several outbreaks occurring at irregular intervals in Africa and Asia between 1960 and 1980 [11]. Following nearly 2 decades of relative quiescence, CHIKV has re-emerged in the last decade, causing major outbreaks in West Africa and among the islands in the Indian Ocean like Madagascar, Comoro, Mayonette and La Réunion. At the same time, CHIKV became entrenched in India and Southeast Asia [12]–[14]. Imported CHIKV cases have reached as far as Japan, China, Taiwan, parts of Europe and the United States of America [15]. As of 2012, the Centers for Disease Control and Prevention (CDC) have listed 46 countries affected by CHIKV (see http://www.cdc.gov/chikungunya/map/index.html). The re-emergence of CHIKV has become a major health concern, making it one of the medically important mosquito-borne viruses of the 21^st^ century [16].

CHIKF first manifests as an acute febrile illness with accompanying headache, rashes, myalgia and polyarthralgia [17]. In some cases, it is followed by chronic pain characterized by persistent arthralgia that can last from weeks to years [18], [19]. The acute symptoms have some similarities with classical dengue, often resulting in misdiagnosis of chikungunya cases in dengue endemic areas in the absence of laboratory confirmation. However, the two can be differentiated since CHIKV infection is more commonly associated with prolonged arthralgia affecting multiple joints, while classical and severe dengue manifest hemorrhagic symptoms [20]. CHIKV infection is generally none life-threatening. Nevertheless, the epidemic in La Réunion that resulted in 265,000 CHIKF cases (roughly one-third of the population) with at least 237 CHIKV-related deaths, and recent reports of more severe clinical manifestation [21], [22] suggest the need to better understand the biology and clinical implications of CHIKV infection. In addition, the global expansion and increased health risks associated with CHIKV infections has prompted the demand for more aggressive efforts to find preventive and therapeutic interventions against this particular disease.

Several chikungunya vaccine strategies have been explored, including inactivated, live attenuated and DNA chimeric vaccines. However, issues concerning safety and efficacy have hampered the progress of current vaccine candidates [23], [24]. Similarly, drugs reported to inhibit CHIKV infection *in cellulo* like chloroquine, ribavirin and arbidol have not shown significant therapeutic benefits in clinical cases [25]–[28]. Recently, cell-based high-throughput assays have been developed to identify potential CHIKV inhibitors. One study reported a focus screen of 356 natural compounds and clinically approved drugs using a CHIKV replicon and a concomitant screen with Semliki Forest Virus (SFV) surrogate infection model [29], while another study screened 3,040 small molecules for inhibitors of CHIKV nsP2 using a novel target-based phenotypic assay approach [30].

High-throughput screening is a technology widely used in today's drug discovery programs that aims to speed up the identification of potentially active substances against various diseases. By using high-throughput assays, a large collection of substances, from small molecules to natural products, can be evaluated for antiviral activity in a relatively short amount of time [31]. The work reported here describes the development of a simple, cell-based high-throughput assay to screen potential CHIKV antivirals. The assay uses resazurin, an oxidized, non-fluorescent blue dye that is converted to the reduced, and highly fluorescent pink-colored resorufin through oxidation-reduction reaction, to measure cellular metabolic activity and cell viability [32], [33]. Using this resazurin reduction assay, a small subset of the BioFocus kinase inhibitor library was screened for compounds that could inhibit CHIKV-associated cell death *in vitro* then confirmed their activities by dose-response curves, virus yield reduction and microneutralization assays. The antiviral activity of the hit compounds was further analyzed using an innovative image-based high content assay to understand the underlying mechanism of action. In addition, the inhibitory properties of the CHIKV primary hits against dengue serotype 2 (DENV2) in a high-content assay screening of the same compound library previously reported [34] was investigated to determine if there are common novel inhibitors between these two unrelated arboviruses.

## Methods

### Cells and Virus

The hepatocarcinoma epithelial cells HuH-7 (JCRB0403), a kind gift from Dr. Katja Fink, was maintained under humidified conditions at 37°C, 5% CO_2_ in RPMI 1640 medium containing 25 mM HEPES (WelGene, South Korea), and supplemented with 10% Fetal Bovine Serum (FBS, Gibco Invitrogen, USA), 100 U/mL penicillin and 100 µg/mL streptomycin (antibiotic solution, Gibco Invitrogen, USA), and passaged every 4–5 days. The mosquito cell line C6/36 *Aedes albopictus* clone (CRL-1660), a generous gift from Dr. Claudia Nuñez Duarte dos Santos, was maintained at 28°C in Leibovitz's L-15 medium (Gibco Invitrogen, USA) supplemented with 5% FBS, 0.26% tryptose phosphate broth (Sigma-Aldrich, USA) and 25 µg/mL gentamicin sulfate (Gibco Invitrogen, USA), and passaged every 3–4 days. The recombinant chikungunya virus with the green fluorescence protein (GFP) gene inserted at the junction region of the viral genome (CHIKV-118-GFP) was kindly provided by Dr. Olivier Schwartz. CHIKV-118-GFP, previously rescued by transfecting the recombinant viral genomic RNA and passaging thrice in BHK21, was passaged twice in C6/36 following the method described elsewhere [35].

### Compound Library and Reference Compounds

A collection of 4,000 small molecules belonging to a kinase-focused chemical library was sourced from BioFocus (Galapagos, Belgium). The reference compounds Bafilomycin A1 (BAF), Chloroquine (CQ), Mycophenolic acid (MPA) and Chlorpromazine (CPZ) were purchased from Sigma-Aldrich (USA). All compounds from the BioFocus kinase inhibitor library, BAF and MPA were dissolved in dimethyl sulfoxide (DMSO, Sigma-Aldrich, USA). CQ was dissolved in Cell-Gro molecular grade water (Mediatech Inc., Manassas, VA).

### Liquid Handling

Automated dispensing of liquid reagents and media containing cells and viruses was performed with the Matrix WellMate (Thermo Fisher Scientific Inc., USA). For the high-throughput screening, compounds from the BioFocus kinase inhibitor library, reference compounds and DMSO vehicle were spotted onto the assay plates using Cybi-Well liquid handler (CyBio, Germany). For plate washing, the 96/384-head BioTek EL406 automated liquid washer/dispenser (BioTek, USA) was used.

### Resazurin Reduction Assay

Resazurin (7-Hydroxy-3*H*-phenoxazin-3-one 10-oxide) was purchased from Sigma-Aldrich (USA) and dissolved in Dulbecco's phosphate-buffered saline (DPBS, pH7.0, WelGene, South Korea). Cell viability was determined by treating with 10 µM resazurin and incubating for 12 hrs at 37°C, 5% CO_2_. Reduction of resazurin to resorufin by cellular enzymes involved in oxidation-reduction reaction was terminated with 3% (w/v) paraformaldehyde (PFA, Sigma-Aldrich, USA) fixative, incubated for 30 min at room temperature. The fluorescence intensity was measured using Victor^3^ V Spectrophotometer (Perkin Elmer, USA) at excitation/emission wavelength of 531/572 nm. To measure the background fluorescence, “EMPTY” wells containing only cell culture medium and treated with resazurin were used. While the fixation step is not critical for the resazurin readout, it allows flexibility in the automation schedule and increase assay robustness by decreasing variability between populations across wells and plates, which may occur when resorufin is further reduced into the colorless and nonfluorescent hydroresorufin [33], [36]. The fluorescence readouts were reported as relative fluorescence units (RFU).

### Virus Titration by Plaque Assay

CHIKV-118-GFP was titrated by plaque assay adapted from previously published method [37]. Briefly, HuH-7 cells grown to 90–95% confluence in 24-well Nunc multidish (Thermo Fisher Scientific Inc., USA) were washed with DPBS and inoculated with 0.2 mL of 10-fold serially diluted CHIKV-118-GFP. After incubating for 2 hrs at 37°C (with rocking every 20 min), excess inoculum was removed and the wells were filled with 0.5 mL overlay medium - RPMI 1640 containing 10% FBS, antibiotic solution and 1.6% carboxymethylcellulose (CMC, Sigma-Aldrich, USA), and incubated for 3 days at 37°C, 5% CO_2_ under humidified conditions. After removal of the overlay medium, the cell monolayers were fixed with 4% PFA at room temperature for 30 min then stained with 0.1% (w/v) crystal violet solution (0.1% w/v crystal violet, 2.5% ethanol in DPBS; crystal violet and ethanol were both purchased from Sigma-Aldrich, USA). Virus plaques were counted and infectious titer was reported as plaque forming units per milliliter (pfu/mL).

### Assay Miniaturization and Optimization

HuH-7 cells were seeded in 384-well *μ* clear-plate black (Greiner Bio-one, Germany) at 5×10^3^ cells per well containing 0.5% DMSO vehicle, and treated with 50 µM CPZ or infected with CHIKV-118-GFP at a multiplicity of infection (M.O.I.) of 0.5 for 24, 48, 72 and 96 hrs at 37°C, 5% CO_2_ under humidified conditions. The wells were fixed with 4% PFA containing 5 µg/mL 4′,6-diamidino-2-phenylindole (DAPI, Sigma-Aldrich, USA) for 30 min at room temperature, then washed twice with 80 µL DPBS and finally filled with 50 µL DPBS. Confocal fluorescence images of DAPI-stained nuclei from 5 different fields of the wells were acquired using the Operetta High Content Imaging System (Perkin Elmer, USA) with 20× objective lens, and analyzed using a customized plug-in on the Image Mining platform software developed in-house. Cell viability of HuH-7 cells using the same experimental conditions described above was determined by resazurin reduction assay.

### Cell and GFP Detection by Imaging

Cell population and CHIKV infection was determined by confocal fluorescence imaging using Operetta and analyzed with the customized plug-in developed within the Image-Mining platform. The latter provides a tool to manipulate and analyze high-content screening data. Within this framework the plugin developed can be used to analyze single or multiple images from well-plate readouts. CHIKV high-content assay analysis is based on image acquired by measuring two different channels: DAPI-stained nuclei and GFP-expressing cells signals emitted at 450 nm and 540 nm, respectively. The cell number was quantified in the DAPI image using a watershed segmentation method [38]. If image is a landscape of peaks and basins, altitude being pixel intensity, then watershed is the flooding of basins with liquid which altitude is progressively increased on the whole image starting from image minima. Each time a local minima is reached a liquid with a new color appears. When 2 liquids meet they do not mix and form a boundary. This flooding process is terminated using a threshold that prevents inclusion of the background in the colored region. Before applying the watershed, unwanted minima due to image noise are filtered out by image blurring. Since nuclei in the DAPI Images are peaks rather than basins, the process is applied to the inverted image. Finally the output is an image where each nuclei region is identified by a unique color. The cell is the number of such defined regions. CHIKV-infected cells were identified by analyzing the degree of overlap between positive GFP signal, selected with a manually defined threshold, and individual nuclei. A cell is considered infected if at least half of its nucleus overlaps with the positive GFP signal. This criterion minimizes false detection due to noise in the GFP signal selection. The percentage of CHIKV-infected cells is defined as the ratio between the detected nuclei overlapping with the positive GFP signal and the total number of detected nuclei.

### Data Normalization, Compound Activity, and Assay Quality Control

The measured RFU values obtained from the resazurin reduction assay was normalized as percent viability using the formula:
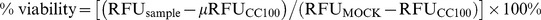
where *μ* represents the mean value, and the RFU_CC100_, RFU_sample_, and RFU_MOCK_ are the measured RFU of the CPZ_50 µM_-treated HuH-7, CHIKV-infected HuH-7 treated with test compound or 0.5% DMSO vehicle, and MOCK-infected HuH-7, respectively. The percent inhibition, which reflects the inhibitory effects of the compounds against CHIKV infection in HuH-7 cells, was calculated using the formula:
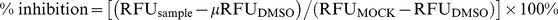
where *μ* represents the mean value, and the RFU_sample_, RFU_DMSO_ and RFU_MOCK_ are the measured RFU of the CHIKV-infected HuH-7 treated with test compounds, 0.5% DMSO vehicle (negative control), and MOCK-infected HuH-7 (positive control), respectively.

### CHIKV High-Throughput Assay Validation

Statistical validation of the CHIKV high-throughput assay was determined using the Z'-factor and coefficient of variation (CV) for the percent inhibition of the positive and negative controls groups, as well as the dose-response curves of reference compounds. The Z'-factor, defined as the degree of separation between positive and negative controls, is calculated using the formula:
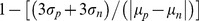
where *μ_p_*, *μ_n_*, *σ_p_* and *σ_n_* represent the means (*μ*) and standard deviations (*σ*) of the positive (*p*) and negative (*n*) controls. A Z'-factor >0.5 between the positive and negative control groups indicates a statistically reliable separation between the positive and negative controls while a CV <10% reflects a low degree of variability within the group [39]. Briefly, freshly trypsinized suspension of HuH-7 cells was inoculated with CHIKV-118-GFP (M.O.I. of 0.5) and dispensed in designated wells of 15 384-well *μ* clear-plate black at 5×10^3^ cells per well. The wells were spotted with 5 µM MPA or 0.5% DMSO vehicle prior to the addition of the cell-virus mixture to simulate the high-throughput screening process. For MOCK-infected HuH-7, cells were mixed with virus medium (Leibovitz's L15 medium supplemented with 1% FBS, 0.26% tryptose phosphate broth and 25 µg/mL Gentamicin sulfate) and dispensed in designated wells as previously stated. The plates were incubated under humidified conditions at 37°C, 5% CO_2_ for 72 hrs, then analyzed by resazurin reduction assay. Scatter-plot distribution and statistical validation of the percent inhibition were generated using TIBCO Spotfire 4.5.0 (TIBCO Software Inc., Somerville, MA). The dose-response curves of the reference compounds was determined by infecting HuH-7 cells with CHIKV-118-GFP (M.O.I. of 0.5) in the presence of various concentrations of BAF (0.4–200 nM), CQ (0.2–100 µM), and MPA (0.2–100 µM) for 72 hrs under humidified conditions at 37°C, 5% CO_2,_ then analyzed by resazurin reduction assay. Compound treatment of MOCK-infected HuH-7 cells were used to measure toxicity. Dose-response curves for the percent inhibition and percent viability were generated using GraphPad Prism 5.04 (GraphPad Software Inc., San Diego, CA). Curve fitting, EC_50_ (concentration showing 50% inhibition) and CC_50_ (concentration showing 50% toxicity) values were elucidated using the software's non-linear regression function: log (agonist) vs. response – variable slope (four parameters) with unconstrained top and bottom values.

### Primary Screening of the BioFocus Kinase Inhibitor Library

The 4,000 compounds screened in this study belongs to the BioFocus kinase inhibitor library - a collection of synthesized small molecules based on predicted interactions with the seven representative subsets of kinases categorized according to protein conformations and ligand binding modes (classical, as well as non-classical) [40]. The chemical library was screened against CHIKV-118-GFP infection in HuH-7 at 10 µM, using 50 µM CPZ as cytotoxicity control, and the MOCK-infected HuH-7 and 0.5% DMSO vehicle as positive and negative controls, respectively. First, the BioFocus compounds, CPZ and DMSO vehicle were spotted onto the 384-well *μ* clear-plate black using the Cybi-Well liquid handler. Second, freshly trypsinized HuH-7 cell suspension was inoculated with CHIKV-118-GFP (M.O.I. of 0.5) or virus medium (for MOCK infection and CPZ_50 µM_ treatment) and dispensed to their designated wells at 5×10^3^ cells per well. The plates were kept under humidified conditions at 37°C, 5% CO_2_ for 72 hrs, then analyzed by resazurin reduction assay. Statistical validation of the positive and negative controls, scatter-plot distribution and histogram of the percent viability were generated using TIBCO Spotfire 4.5.0. Primary hits were selected using a statistical cut-off based on the percent viability of the DMSO vehicle control. The statistical cut-off value was determined using the formula:
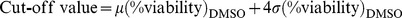
where *μ*(%viability)_DMSO_ and 4*σ* (%viability)_DMSO_ are the mean and 4 standard deviations of the DMSO vehicle control, respectively.

### Counter-screening against Dengue Virus Serotype 2 (DENV2)

The inhibitory property of the CHIKV primary screening hits against DENV2 infection was investigated by retrieving the results from the a high-content, high-throughput screening of the BioFocus kinase inhibitor library against DENV2 infection in Huh-7.5 cells (a derivative of the HuH-7) that was reported previously [34]. In that screening campaign, Huh-7.5 (PTA-8561, U.S. Patent Number 7455969) was inoculated with DENV2 (BR DEN2 01-01, GenBank JX073928) (M.O.I. 0.5) in the presence of 10 µM compounds and incubated under humidified conditions at 37°C, 5% CO_2_ for 72 hrs. Percent infection and cell number was determined using an image-based immunofluorescence detection of dengue E protein in infected cells by flavivirus group-specific αE monoclonal antibody 4G2 and nuclei staining with DAPI, and analyzed using another customized plug-in of the Image Mining platform. Compounds that showed DENV2 inhibition ≥80% and exhibited ≤50% toxicity based on cell number at 10 µM were considered as positive hits.

### Hit Confirmation by Dose-Response Curves

The antiviral activity and toxicity of the primary hits from the CHIKV high-throughput screening of the BioFocus kinase inhibitor library was confirmed by dose-response curves. For measuring antiviral activity, HuH-7 cells were mixed with CHIKV-118-GFP (M.O.I. 0.5) and seeded at 5×10^3^ cells per well in 384-well *μ* clear-plate black spotted with 2-fold serial dilution of the hit compounds (0.1–50 µM). For measuring toxicity, MOCK-infected HuH-7 cells were seeded in 384-well *μ* clear-plate black containing the hit compounds prepared as above. The plates were kept under humidified conditions at 37°C, 5% CO_2_ for 72 hrs, then analyzed by resazurin reduction assay. The dose-response curves, EC_50_ and CC_50_ based on percent inhibition and percent viability was generated with GraphPad Prism 5.04 software using the curve fitting parameters stated previously. The *Selectivity Index* (*SI*), a dimensionless value that indicates the magnitude between cytotoxic and effective concentrations of the compound, was calculated using the formula: *SI* = CC_50_/EC_50_.

### Counter-screening for Resazurin Fluorescence

Some inhibitors of mitochondrial activity have been shown to catalyze the reduction of resazurin in the medium to a certain extent [32]. To confirm that the observed inhibitory property of the hit compounds against CHIKV-induced cell death was not a result of the compound's reduction of resazurin, varying concentrations (1.5 µM–50 µM) of the hit compounds were added into the culture medium without cells then analyzed by resazurin reduction assay.

### Structural Analysis of Hit Compounds

Cluster analysis was done using a molecule-clustering module from Pipeline Pilot (Accelrys Software Inc., San Diego, CA, USA). Structural relationship among the primary hit compounds was analyzed using the Tanimoto coefficient structural similarity [41]. The core scaffolds of hit compounds that exhibited anti-CHIKV activity were selected for structural analysis.

### Virus Yield Reduction Assay

The effect of the hit compounds on the production of infectious virions was determined by plaque assay. Briefly, HuH-7 cells grown to confluence in 96-well Costar flat bottom plates (Corning, USA) were treated with 50 µM MPA, 50 µM CQ, 20 µM hit compounds or 0.5% DMSO vehicle, then inoculated with CHIKV-118-GFP (M.O.I. of 0.5). After 1 hour, excess inoculum was removed and the wells were replenished with fresh culture medium containing the same amount of the compounds and incubated for 24 hrs at 37°C, 5% CO_2_ under humidified conditions. The titer of infectious progeny virions released into the culture medium was determined by plaque assay.

### Microneutralization Assay

The microneutralization assay was performed to assess the protection conferred by the hit compounds against CHIKV-induced cytopathic effect (CPE), adapting a previously described method used for evaluating protective antibodies [42]. Briefly, HuH-7 cells were seeded in 96-well Costar flat bottom plates at 5×10^4^ cells per well and kept under humidified conditions at 37°C, 5% CO_2_ for 24 hrs. Two-fold serial dilution of the hits compounds (0.2–100 µM) were added designated wells, then inoculated with 50 pfu CHIKV-118-GFP and incubated for 1 hr at 37°C. Excess inoculum was removed from the wells and replenished with fresh culture medium containing the same amount of the compounds. The culture was incubated for 72 hrs under humidified conditions at 37°C, 5% CO_2_. To visualize the damage to the cell monolayer caused by CHIKV-induced CPE, wells were fixed with 4% PFA for 30 min at room temperature, then stained with 0.1% crystal violet solution. The endpoint was defined as the lowest concentration of the compound that inhibited CHIKV-induced CPE. The assay was performed in quadruplicates.

### Image-Based High-Content Assay of CHIKV Infectivity

CHIKV-118-GFP infection of HuH-7 in the presence of the hit compounds for 24 hours was analyzed by image-based high-content assay. HuH-7 cells were seeded in 96-well *μ* clear-plate black (Greiner Bio-one, Germany) at 5×10^4^ cells per well and kept under humidified conditions at 37°C, 5% CO_2_ for 24 hrs. CHIKV-118-GFP was inoculated at a M.O.I. of 0.5 in the presence of 50 µM MPA, 50 µM CQ, 20 µM hit compounds or 0.5% DMSO vehicle and incubated under humidified conditions for 24 hrs at 37°C, 5% CO_2_. Cells were fixed and stained with 4% PFA containing DAPI (5 µg/mL) for 30 min at room temperature, then washed thrice with DPBS. Confocal fluorescence images from 10 different areas of the well were acquired using Operetta (20× objective lens) and analyzed by the customized plug-in software in the Image Mining platform. The assay was performed in quadruplicate.

## Results

### Development of the Cell-Based Chikungunya High-Throughput Assay Using Resazurin

**Figure 1:**
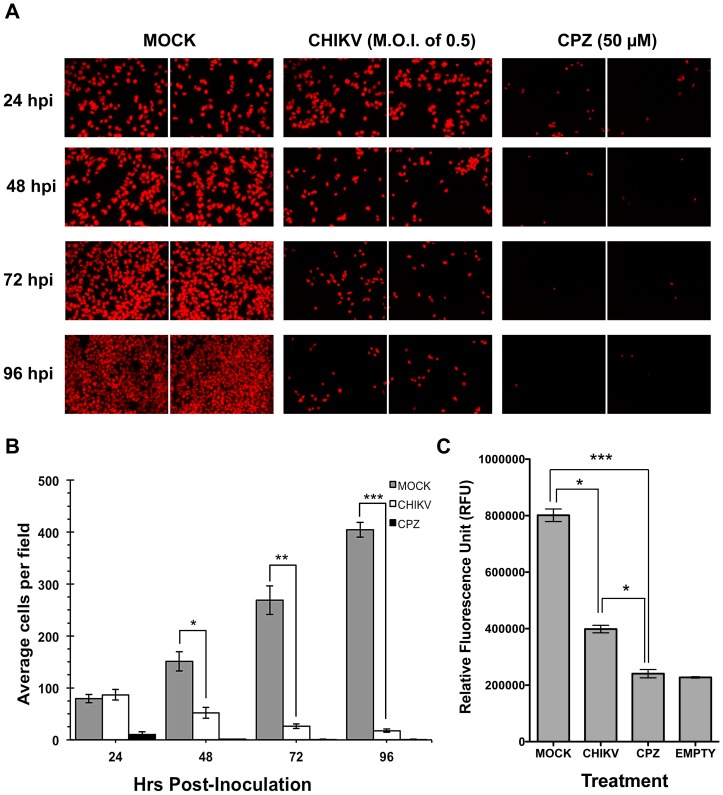
Correlation of CHIKV-infection with HuH-7 cell viability. DAPI-stained nuclei of MOCK-infected, CHIKV-infected and CPZ-treated HuH-7 cells observed using Operetta at 20× magnification (A). Average number of cells per field based on detected nuclei (B). RFU readout after resazurin treatment at 72 hpi (C). (false-color imaging were applied on panel A).

We developed a cell-based high-throughput assay system using the hepatocarcinoma HuH-7 cell line and resazurin to assess CHIKV-associated cell death. HuH-7 was selected as the target cells based on previous reports demonstrating its high susceptibility to CHIKV infection [43], [44]. Cell viability was determined by measuring the reduction of resazurin to the highly fluorescent resorufin by metabolically active and viable cells [33]. Using an initial seeding density for HuH-7 at 5×10^3^ cells per well, the effect of CHIKV-118-GFP infection (M.O.I. of 0.5) on cell number and cell viability was measured at 24, 48, 72 and 96 hrs post-infection (hpi), with 50 µM CPZ as a control cytotoxic compound. Cell number was quantified by staining the nuclei with DAPI and analyzed using a customized plug-in of the Image Mining platform while cell viability was measured by resazurin reduction assay. Figure 1A and 1B show the number of detected nuclei at 24, 48, 72 and 96 hpi. CHIKV-118-GFP infection of HuH-7 cells started showing significant reduction in cell number beginning from 48 hpi compared with MOCK-infection (*P<0.0001*). The percent reduction in the average number of cells resulting from CHIKV-118-GFP infection was 65%, 90%, and 95% at 48, 72, and 96 hpi, respectively. In comparison, treatment with 50 µM CPZ dramatically reduced the cell number by 86% after 24 hrs, and >99.9% after 48, 72, and 96 hrs. Based on the resazurin reduction assay, CHIKV-infected and CPZ-treated HuH-7 cells showed significant reduction in cell viability after 72 hpi, as shown by their lower fluorescence readout (Figure 1C). Treatment of HuH-7 cells with 50 µM CPZ resulted in a measured fluorescence readout (240,842±14,730 RFU) that was nearly identical with EMPTY wells (227,447±2,446 RFU), suggesting complete abolition of metabolic activity. In comparison, CHIKV-infected HuH-7 showed higher fluorescence readout (398,579±13,294 RFU), but was still significantly lower than that of MOCK-infected HuH-7 (801,587±22,230 RFU). It has been reported previously that short-term resazurin reduction occurs in dying cells caused by the production of free and unpaired electrons [45] While a good fluorescent signal can be achieved from MOCK-infected cells, distinguishable from that of CPZ-treated cells and EMPTY wells, within 6 hours after treatment with resazurin, the 12-hour treatment was necessary to have a clear separation of fluorescent signals between MOCK-infected and CHIKV-infected cells (data not shown).

### Validation of CHIKV High-Throughput Assay

**Figure 2:**
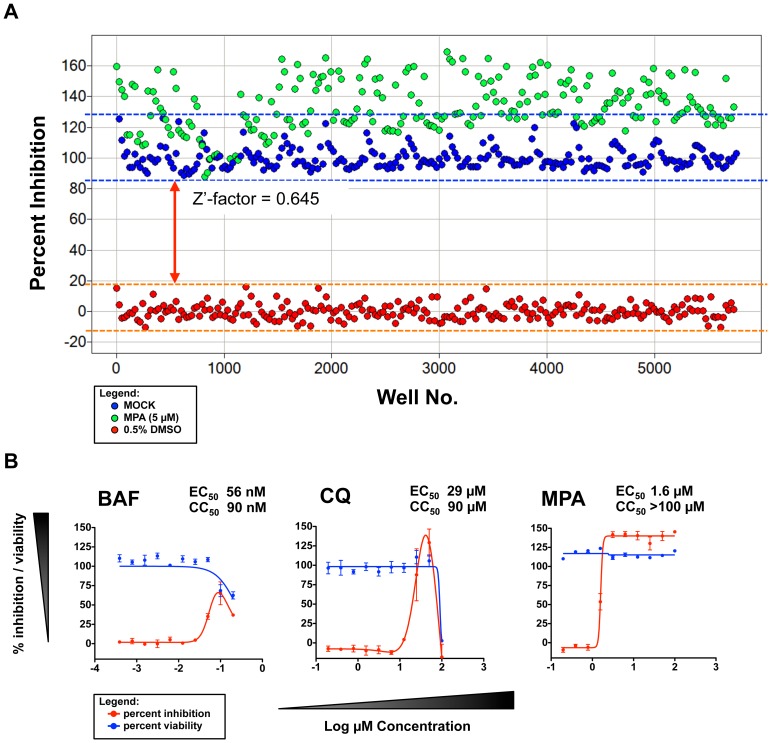
Validation of the CHIKV high-throughput assay. Scatter plot and calculated Z'-factor of the resazurin reduction assay for CHIKV high-throughput screening. Dots represent wells with the following treatment: MOCK-infected HuH-7 (blue), CHIKV-infected HuH-7 with 0.5% DMSO vehicle (red), and 5 µM MPA (green). Area under the blue and orange dotted lines represent the variability of the measured percent inhibition for CHIKV-infection and MOCK infection controls, respectively. Arrow represents the degree of separation (Z'-factor) between MOCK and CHIKV infection controls (A). Dose-response curves of BAF, CQ, and MPA anti-CHIKV activity (red) and their effect on HuH-7 cell viability (blue) (B).

The use of resazurin reduction assay for high-throughput screening of CHIKV inhibitors was evaluated using the Z'-factors for the “percent inhibition” against CHIKV-infection between the 0.5% DMSO vehicle and 5 µM MPA-treated groups and between 0.5% DMSO vehicle and MOCK-infected groups. Figure 2A shows the scatter-plot distribution of the percent inhibition for the 0.5% DMSO vehicle, 5 µM MPA and MOCK-infected groups. The average Z'-factor between the CHIKV-infected 0.5% DMSO vehicle and 5 µM MPA-treated groups in the 15 384-well test plates was 0.578±0.075 (Z'-factors ranged from 0.460 to 0.703) while the average Z'-factor between the CHIKV-infected 0.5% DMSO vehicle and MOCK-infected groups was 0.645±0.059 (Z' factors ranged from 0.544 to 0.724). The RFU of the 0.5% DMSO vehicle negative control group showed a higher degree of variability (CV = 13.9±3.3%) compared with those of the positive control groups −5 µM MPA-treated (CV = 8.1±1.6%) and MOCK-infected (CV = 5.1±1.3%). While the 0.5% DMSO vehicle control group showed a CV slightly higher than 10%, the Z'-factors between the MOCK-infected and 0.5% DMSO vehicle control groups were >0.5 in all 15 test plates, indicating a reasonable separation between positive and negative controls. These findings demonstrate the reliability of the resazurin reduction assay for use in the CHIKV high-throughput screening. Figure 2B shows the activity and toxicity of 3 reference compounds (bafilomycin A1, chloroquine and mycophenolic acid) against CHIKV infection in HuH-7. BAF showed an EC_50_ value of 56 nM. Since BAF did not exhibit >50% toxicity at the highest concentration tested (200 nM), a projected CC_50_ value 237 nM was extrapolated based on the trend of the percent viability curve. For CQ, the EC_50_ and CC_50_ values were determined at 29 µM and 90 µM, respectively. A bell-shaped curve was observed for the percent inhibition as the concentration of CQ approached 100 µM and coincided with the steep decline in percent viability, indicating high toxicity at 100 µM. MPA showed an EC_50_ value of 1.6 µM, with no observable cytotoxicity at the highest concentration tested (100 µM). The antiviral activity of MPA determined by the resazurin reduction assay was within the range of the 50% inhibitory concentration of MPA previously reported (1.5 µM–4.1 µM) [29], while the measured antiviral activity and toxicity of BAF and CQ were within 3-fold of the observed inhibitory property from previously published reports [46], [47]. Interestingly, MPA treatment of CHIKV-infected HuH-7 cells at concentrations ≥3.25 µM resulted in higher fluorescence readout compared with non-infected HuH-7 treated with the same amount of the compound. The underlying mechanism for this observed phenomenon has not been elucidated.

### Screening of the BioFocus Kinase Inhibitor Library against CHIKV

**Figure 3:**
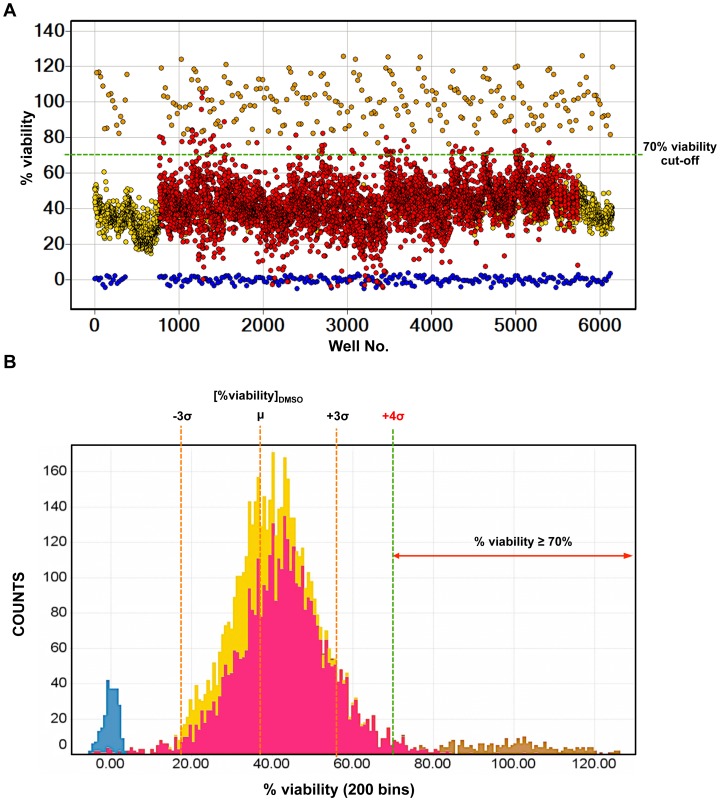
CHIKV high-throughput screening of the BioFocus kinase inhibitor Library. Scatter plot distribution showing the results of the CHIKV high-throughput screening of the BioFocus kinase inhibitor library using resazurin reduction assay. Dots represent the normalized RFU of MOCK-infected HuH-7 with 0.5% DMSO vehicle (brown), CHIKV-infected HuH-7 with 0.5% DMSO vehicle (yellow), CHIKV-infected HuH-7 with 10 µM BioFocus compounds (red) or MOCK-infected HuH-7 with 50 µM CPZ (blue) (A). Histogram showing the distribution of MOCK-infected HuH-7 (brown), CHIKV-infected HuH-7 (yellow), CHIKV-infected HuH-7 with 10 µM BioFocus compounds (red) or MOCK-infected HuH-7 with 50 µM CPZ (blue) (B). The orange dotted lines represent 99.7% (μ±3σ) of the CHIKV-infected HuH-7 wells, while the green dotted lines represent the statistical cut-off (μ+4σ of the DMSO vehicle control) used for selecting primary hits (≥70% viability).

The 4,000 compound subset belonging to the BioFocus kinase inhibitor library was screened for potential CHIKV antivirals using the resazurin reduction assay which detects the highly fluorescent resorufin resulting from the reduction of resazurin by metabolically active and viable cells (Figure S1). All the compounds from the BioFocus library were screened at 10 µM in 0.5% DMSO. The measured RFU from each compound treatment was normalized as percent viability using the average RFU of MOCK-infected and CPZ_50 µM_-treated HuH-7. Figures 3A shows the overview of the results of the CHIKV high-throughput assay screening of the BioFocus kinase inhibitor library, with the percent viability of each treatment represented by colored dots. A histogram of the percent viability showing the frequency distribution for each treatment (BioFocus compounds, CPZ, DMSO and MOCK) is shown in Figure 3B. The average percent viability of CHIKV-infected HuH-7 cells treated with 0.5% DMSO vehicle from plate to plate ranges between 33.99%±7.51% and 38.36%±7.39%. Using the upper limit of this range, the computed statistical cut-off (μ+4σ) was 67.92% viability. Based on this computed value, statistical cut-off was rounded off to 70% viability. A total of 72 compounds from the BioFocus kinase inhibitor library showed ≥70% viability in the CHIKV high-throughput screening and were selected as primary hits, giving a hit rate of 1.8%. The computed percent inhibition of these 72 hit compounds against CHIKV-associated cell death was >50% (Figure S2). Among the primary hits identified against CHIKV, compound CND1611 (59.85% inhibition) was previously identified as a primary hit against DENV2 (100% inhibition, 44.57% toxicity at 10 µM) in the dengue high-content assay screening of the BioFocus kinase inhibitor library. However, follow-up on this compound was discontinued after hit confirmation by dose-response curve did not reveal any clear antiviral activities against CHIKV (data not shown). Compound CND3514, which was later confirmed to exhibit antiviral activity against CHIKV, had only shown moderate inhibitory property against DENV2 (67.87% inhibition, 39.37% toxicity at 10 µM).

### Dose-Response Curves Confirm Antiviral Activity of CHIKV Primary Hits

The antiviral activity of the 72 primary hits in the CHIKV high-throughput screening was confirmed by measuring their inhibitory property and toxicity at different concentrations (0.1 µM–50 µM) using the resazurin reduction assay. Among these, 6 hit compounds (CND0335, CND0364, CND0366, CND0415, CND0545 and CND3514) inhibited CHIKV-associated cell death in a dose-dependent manner (Figure S3). However, the dose-response curves for most of these confirmed hits behaved differently from that of mycophenolic acid. With the exception of compound CND0366, the inhibitory activities of the other 5 confirmed hits were observed to plateau between 55.32% and 83.35%. None of the confirmed hits exhibited significant toxicity in HuH-7 cells at the highest concentration tested. Conversely, some showed an increase in viability at higher concentrations based on the resazurin reduction readout. A counter-screen to determine if the compounds can contribute to the increased readout was carried out by mixing the compounds with resazurin in the medium. As shown in the Table S1, there was no increase in the RFU readout by the compounds alone, thus ruling out the direct reaction of resazurin with these particular compounds. The 6 confirmed hits showed EC_50_ values ranging from 2.2 µM to 7.1 µM based on the 50% inhibition relative to the maximum inhibition achieved by each compound. The EC_50_, CC_50_ and *SI* values of the 6 confirmed hit compounds are summarized in Table 1.

**Table 1:** Activity profile of the 6 confirmed hit compounds against CHIKV based on resazurin reduction assay.

Compound ID	Core scaffold	EC_50_ a (µM)	CC_50_ b (µM)	*SI* c
CND0335	benzofuran	3.3	>50	>15
CND0415	benzofuran	6.2	>50	>8.1
CND0364	benzofuran	6.2	>50	>8.1
CND0366	benzofuran	7.1	>50	>7.0
CND0545	pyrrolopyridine	5.6	>50	>8.9
CND3514	thiazol-carboxamide	2.2	>50	>22.7

a EC_50_ is the concentration showing 50% inhibition relative to the maximum inhibition achieved.

b CC_50_ is the concentration resulting in 50% toxicity in HuH-7 cells.

c *SI* is determined by the formula: CC_50_/EC_50_. Values indicated are the minimum *SI* based on the highest concentration of compound tested.

### Chemical Structures of Anti-CHIKV Compounds

**Figure 4:**
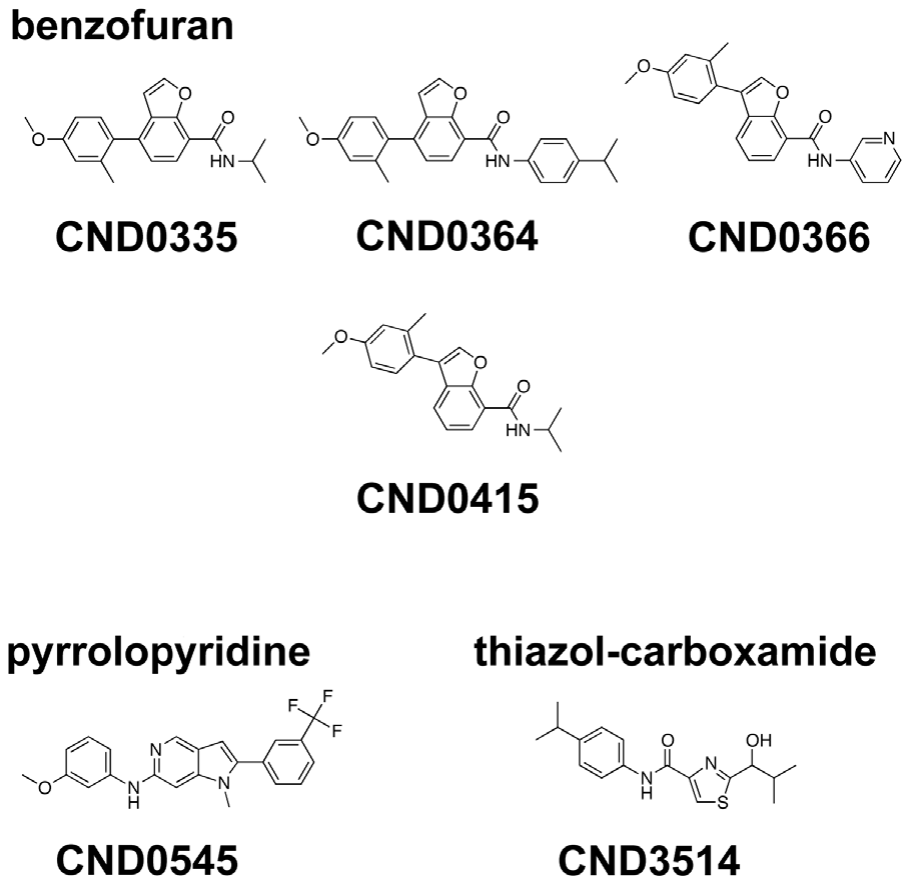
Chemical structures of the 6 hit compounds exhibiting anti-CHIKV activities.

The chemical structures of the 6 confirmed hit compounds are shown in Figure 4. Based on the structural analysis using the molecule-clustering module from Pipeline Pilot, 4 of the compounds - CND0335, CND0364, CND0366, CND0415 share a benzofuran scaffold, with a common substitution of 4-methoxy-2-methylphenyl at the 3- or 4- positions in the benzofuran core plus amide linker with hydrophobic groups at the 7- position. Two other compounds, CND0545 and CND3514, are singletons having a pyrrolopyridine or a thiazol-4-carboxamide base scaffold, respectively.

### Development of the Image-Based High-Content Assay for CHIKV-118-GFP Infection

**Figure 5:**
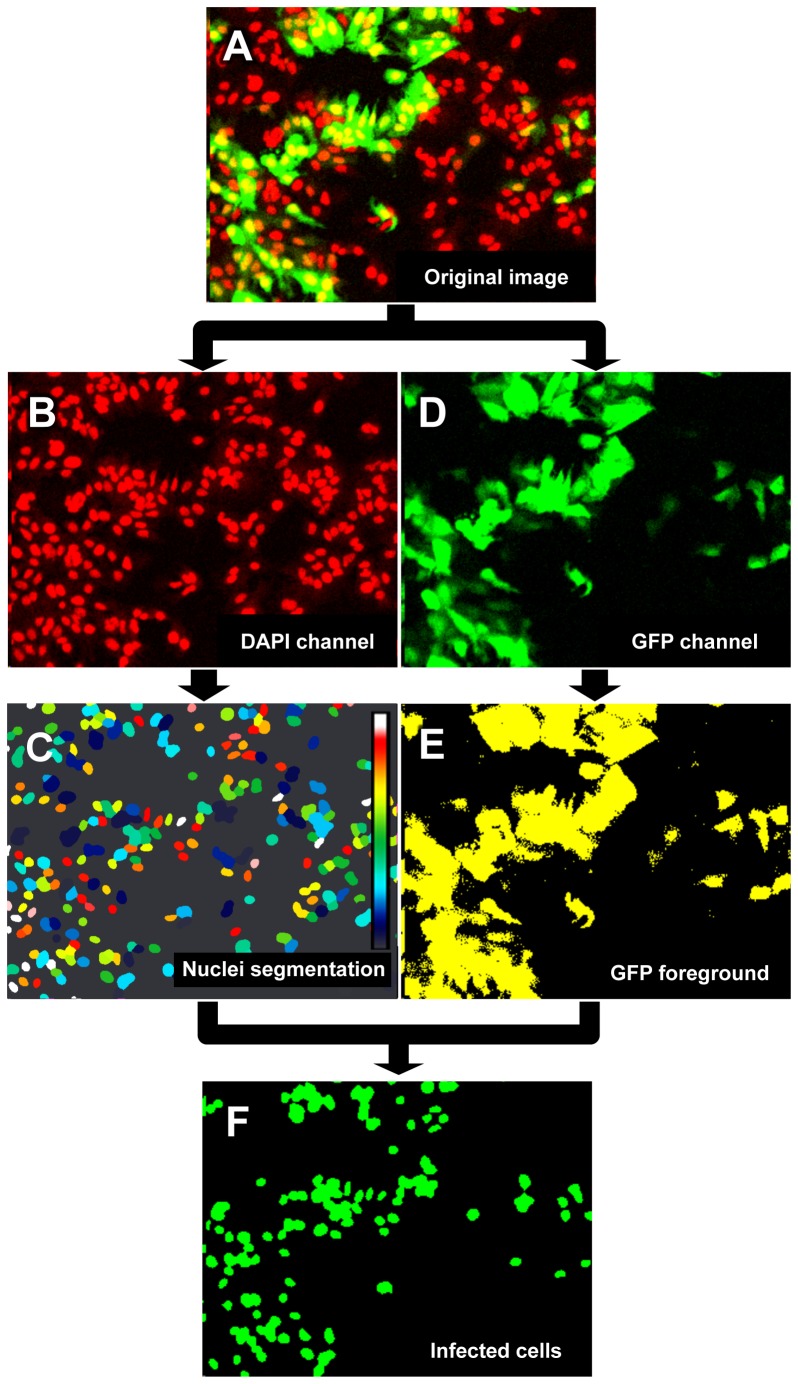
Quantification of CHIKV-118-GFP-infected cells by imaging. Acquired confocal images of CHIKV-118-GFP infection in HuH-7 cells analyzed using the customized plugin in the Image Mining platform. From the merged image (A), the regions of individual nuclei from the DAPI channel (B) are defined using a watershed algorithm (C). The resulting output shows each individual nucleus with distinct color (A color map is drawn in frame C). A positive signal from the GFP channel (D) is determined using a threshold value to generate the GFP foreground (E). CHIKV-infected cells are identified by the overlap between nuclei identified in C and GFP foreground in E (F) and the percentage of nuclei of infected cells is defined by the ratio between nuclei in F and C. (false-color imaging were applied on panels A, B, and C).

CHIKV-118-GFP is a recombinant virus that has a GFP reporter gene with its own 26S subgenomic promoter inserted into the viral genomic RNA. During CHIKV-118-GFP infection, the GFP reporter gene is transcribed as a separate subgenomic mRNA from the 26S subgenomic mRNA by utilizing its own 26S subgenomic promoter. Expression of the green fluorescence protein indicates infection of the host cell with CHIKV and the proper functioning of the viral replicase [48]. GFP expression in CHIKV-infected cells and DAPI-stained cell nuclei are detected by confocal fluorescence imaging using Operetta. A customized plug-in in the Image Mining platform analyzes the GFP and DAPI channels of the acquired confocal fluorescence images using a watershed segmentation method [38] and generates numerical data such as the total number of nuclei and percentage of cells expressing GFP (Figure 5). Using this image-based high-content assay, more than 50% infected cells were detected at 18 hpi when HuH-7 cells are inoculated with CHIKV-118-GFP at a M.O.I. ≥0.5, and more than 98% infected cells within 24 hpi (Figure S4). Based from these results, the image-based high-content assay was used to evaluate the antiviral activity of the 6 confirmed hit compounds against CHIKV infection in HuH-7.

### Hit Compounds Inhibit CHIKV by Interfering with Virus-Induce CPE

**Figure 6:**
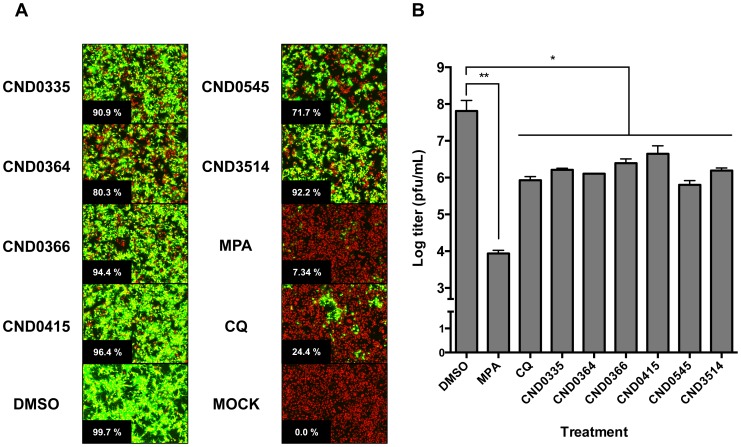
Inhibition of virus infectivity. Image analysis on CHIKV-118-GFP infection of HuH-7 in the presence of 20 µM hit compounds, 50 µM MPA or 50 µM CQ. The percentages of infected cells are indicated on the lower right of each image (A). Production of infectious virus particles from the CHIKV-118-GFP-infected HuH-7 in the presence of 20 µM hit compounds, 50 µM MPA and 50 µM CQ (B). DMSO – CHIKV-118-GFP infection control, MOCK – non-infected control, MPA – mycophenolic acid, CQ – chloroquine. (false-color imaging were applied on panel A).

**Figure 7:**
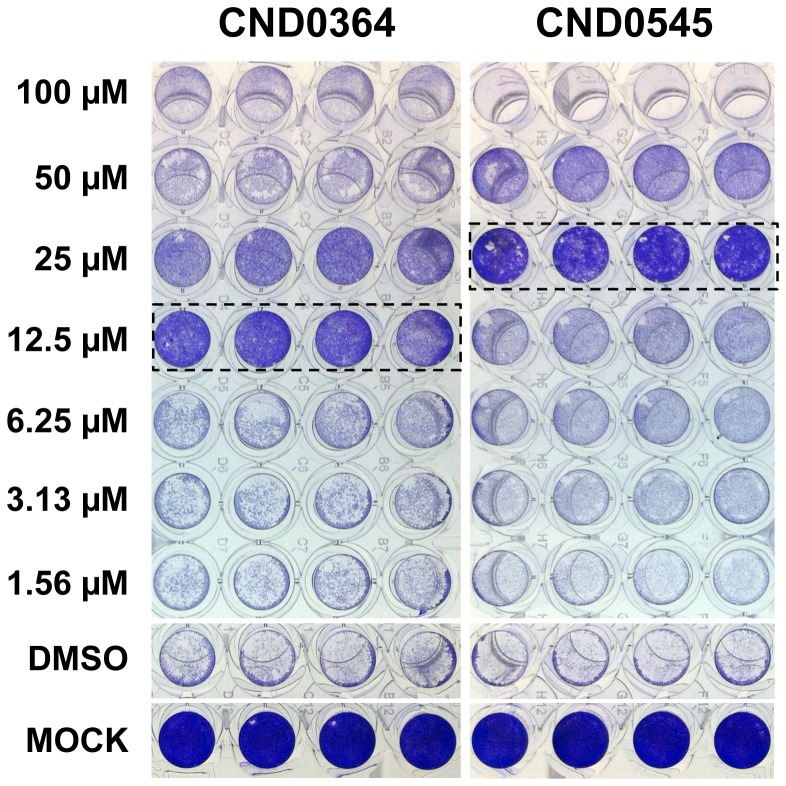
Virus neutralization assay. Confluent monolayer of HuH-7 cells were infected with 50 pfu CHIKV-118-GFP in the presence of compound CND0364 or CND0545 at various concentrations for 72 hrs, and then stained with 0.1% crystal violet solution to observe clearance of the cell monolayer caused by virus infection. Dotted boxes indicate concentration of compounds that exhibit protection against CHIKV-induced CPE. (DMSO – infection control, MOCK – negative control).

The effect of the 6 hit compounds on CHIKV-118-GFP infection of HuH-7 cells was evaluated by quantifying GFP-expressing GFP and comparing cell morphology during infection. The inhibitory properties of two reference compounds and the 6 confirmed hit compounds against CHIKV-118-GFP were evaluated by image analysis using the customized plug-in on the Image Mining platform (see Figure 6A). Chloroquine, a lysosomotropic agent that blocks viral entry by preventing pH dependent fusion [49], inhibited CHIKV-118-GFP infection by 75.6% at 50 µM. Mycophenolic acid, an inhibitor of GMP synthesis that results in decreased synthesis of RNA and DNA [50], inhibited more than 92% of infection at 50 µM. In contrast to CQ and MPA, none of the 6 hit compounds exhibited significant inhibition of CHIKV-118-GFP infection in HuH-7. The benzofuran and thiazol-4-carboxamide compounds showed <10% inhibition at 20 µM, with the exception of CND0364 (19.7% inhibition at 20 µM). Also, the pyrrolopyridine compound CND0545 only inhibited 28.3% of infection at 20 µM. However, it was observed that in the presence of the 6 hit compounds, the morphology of CHIKV-infected cells showed less apoptotic blebs compared with those treated with the DMSO vehicle control. Furthermore, the culture supernatant of CHIKV-infected HuH-7 cells showed a 10- to 100-folds decrease (1.16–2.01 log titer reduction) in viral titer when treated with the hit compounds compared to the DMSO vehicle control (Figure 6B). Interestingly, the reduction in viral titers resulting from the treatment of 6 hit compounds was comparable with that of chloroquine treatment (1.88 log titer reduction). Compounds CND0364 and CND0545, which exhibited the highest reduction in viral titers (1.69 log and 2.01 log titer reduction, respectively), inhibited the clearing of the cell monolayer caused by CHIKV-induced CPE at 12.5 µM and 25 µM, respectively in the microneutralization assay (Figure 7).

## Discussion

In recent years, the number and distribution of CHIKV cases have dramatically increased, culminating in the most devastating outbreak recorded between 2005–2006 in La Réunion [14], [22]. The rising incidence of chikungunya outbreaks has spurned renewed efforts to find an effective vaccine to address this potential emerging epidemic [51]. Ironically, very few studies focusing on CHIKV antiviral drugs have been reported until recently. The use of chloroquine, a quinolone-containing drug used as an antimalarial drug [52], for therapeutic intervention of CHIKV-associated arthralgia was previously suggested [53]. While chloroquine showed inhibitory properties against CHIKV infection in vitro, it has a narrow selectivity index in cell cultures [46]. Furthermore, double-blind placebo-controlled randomized trial for treatment of acute chikungunya infections in La Réunion with chloriquine did not show any significant benefits in the use of this drug [54]. Ribavirin, a nucleoside analogue prodrug that has been widely used as an antiviral for several DNA and RNA viruses [55], was also proposed as a therapeutic drug to treat chikungunya-associated arthritis [28]. However, no further follow-up studies were pursued. Another antiviral drug originally used for treatment of influenza and other respiratory viruses - arbidol - was also reported to have potent antiviral properties against CHIKV in vitro, and was found to bind to the E2 domain of the viral envelope protein and interfere with viral attachment on host cell receptor [27]. Recently, a bioactivity screening method to identify inhibitors of alphavirus entry and replication was reported [29]. The assays performed in this particular screening involved the use of a non-cytotoxic CHIKV replicon expressing *EGFP* and *Rluc* and a SFV surrogate model. The study reported several active 5,7-dihydroxyflavones (e.g. apigenin, chrysin, naringenin and silybin) from a collection of natural products that suppresses CHIKV and SFV replication, suggesting these molecules may be good antiviral candidates against alphavirus infections. Another cell-based phenotypic assay that identify inhibitors of CHIKV nsP2-mediated transcriptional shutoff was also recently reported [30].

Here we reported the development of a simple cell-based high-throughput assay using resazurin for the screening of bioactive molecules against CHIKV. Resazurin has been used mainly for detecting microbial contamination in milk and other food products for the past 70 years [56], [57]. However in the last 20 years, several resazurin-based assays have been developed for screening or evaluation of active molecules against microbial pathogens like *Mycobacterium sp.* [58], [59], *Trichomonas vaginalis* [60], *Trypanosoma brucei* [61], *Leishmania sp.* [36], *Aspergillus fumigatus* [62], as well as cancer [63], [64] and *Clostridium perfringens* ε-toxin [65]. The resazurin reduction assay assesses cell viability by measuring the metabolic capacity of these cells to reduce resazurin (also known as Alamar blue) to the highly fluorescent resorufin. It has been shown that there is a positive linear correlation between metabolic reduction of resazurin and the number of viable cells [33], [66]. In viable eukaryotic cells, the conversion of resazurin to resorufin is facilitated by mitochondrial reductases and diaphorases such as dihydrolipoamine dehydrogenase (EC 1.8.1.4,), NAD(P)H:quinone oxidoreductase (EC 1.6.99.2) and flavin reductase (EC 1.6.99.1) found in the mitochondria or cytoplasm [33], [67]. CHIKV causes highly cytopathic infection in a wide variety of cells of vertebrate origin, resulting in rapid death of infected cells by apoptosis [46]. Thus, the resazurin reduction assay was used to identify potential CHIKV inhibitors by measuring cell viability, which serves as an indicator of inhibitory property against CHIKV-associated cell death. Unlike the non-cytotoxic CHIKV replicon used for the bioactive screening, our CHIKV high-throughput assay uses an infectious recombinant CHIKV (CHIKV-118-GFP) that is highly cytopathic in HuH-7 cells. Screening the 4,000 compound subset of the BioFocus kinase inhibitor library using the resazurin reduction assay identified 6 active compounds that inhibit CHIKV-associated cell death – 4 having a benzofuran core scaffold, 1 with a pyrrolopyridine scaffold, and 1 with a thiazol-carboxamide scaffold – with EC_50_ in the single-digit micromolar range. However, it was observed that the inhibitory properties of the active compounds at the highest concentration tested (50 µM) were not enough for the metabolic activity of the host cell infected with CHIKV to reach the same level as that of non-infected cells. Moreover, the percentage of CHIKV-infected cells in the image-based high-content assay of CHIKV-118-GFP infection did not diminish significantly even in the presence of 20 µM concentration of the active compounds, an indication that the their activity have little or no effect in the viral entry process or viral replication machinery. Interestingly, despite the high percentage of CHIKV-infected cells, treatment with 20 µM of the active compounds reduced viral titers up to 100-fold, suggesting that their activity likely targets the later stages of viral infection (*i.e.*, virus assembly and release).

**Figure 8:**
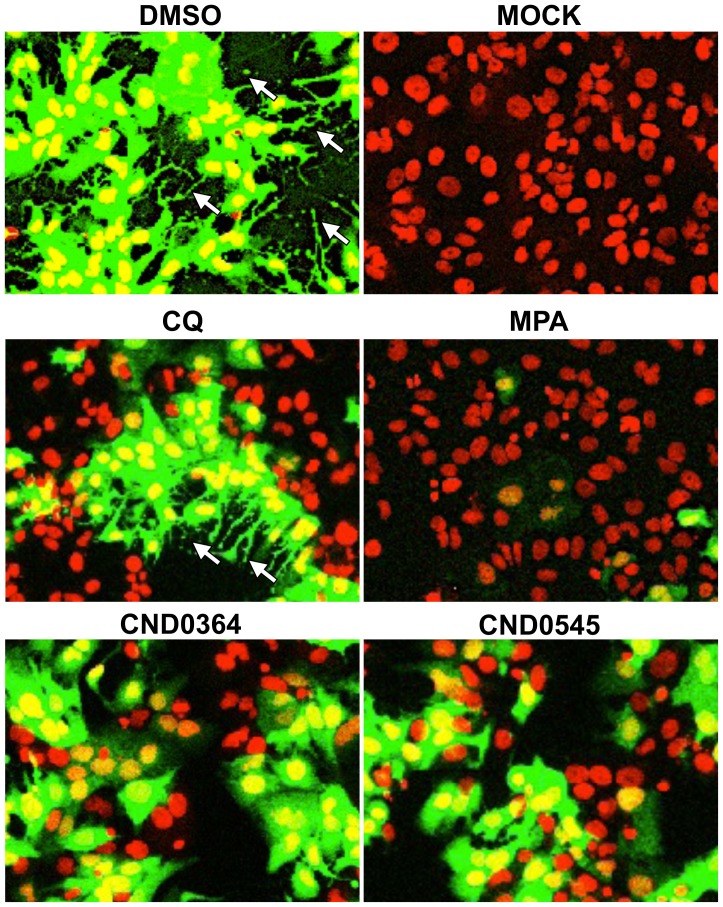
Inhibition of CHIKV-associated apoptotic bodies. HuH-7 cells infected with CHIKV-118-GFP expressing GFP at 24 hpi. The number of observed apoptotic blebs associated with CHIKV infection (marked by white arrows) were significantly lower when treated with the reference compound MPA and hit compounds CND0364 and CND0545. In contrast, apoptotic blebs were still prominent in CHIKV-infected HuH-7 cells in the presence of CQ. (false-color imaging were applied).

Several factors have been implicated in the persistence of arthralgia after CHIKV infection. After CHIKV replicates in the liver, it targets muscle satellite cells, as well as other cells and tissues like muscle, joint and fibroblasts [68], [69]. Persistence of CHIKV infection in microglial cells and perivascular synovial macrophages have been shown to trigger host-derived inflammatory cytokine responses that contribute directly to synovial tissue damage [70]–[73]. Viral persistence has been attributed to the ability of the virus to evade the host immune response by different mechanisms. Recently, a novel mechanism of immune evasion by CHIKV involving the host cell's apoptotic machinery has been described. CHIKV particles have been shown to hide in apoptotic blebs of infected cells and invade neighboring cells or cells that are otherwise refractory to CHIKV alone, such as macrophages, through phagocytosis of these apoptotic bodies [6], [74]. The two active compounds that yielded the highest reduction in CHIKV viral titers (CND0364 and CND0545) were shown to inhibit CHIKV-induced CPE in the microneutralization assay. The phenotypic characteristic of CHIKV-118-GFP infected HuH-7 cells treated with the benzofuran compound CND0364 and pyrrolopyridine compound CND0545 showed a dramatic reduction in the formation of apoptotic blebs compared with the DMSO vehicle control treatment, as well as chloroquine treatment (Figure 8). These findings suggest that the benzofuran and pyrrolyridine compounds impede the efficient dissemination of CHIKV into the neighboring cells by interfering with the virus-induced apoptotic machinery.

The exact role of cellular kinases in promoting CHIKV-induced apoptosis remains poorly understood. It has been reported that CHIKV dsRNA activates dsRNA-dependent protein kinase (PKR). PKR phosphorylates translation initiation factor 2-alpha (eIF2α) and plays a role, albeit not essential, in CHIKV-associated cellular translational shutoff [75]. Ironically, it has been shown recently that CHIKV nsP4 suppresses eIF2α phosphorylation that regulates the PKR-like ER resident kinase (PERK) pathway [76], an ER stress pathway implicated in mediating cell apoptosis [77]. CHIKV has also been reported to trigger an autophagic process that promotes viral replication [78]. However, CHIKV-induced autophagy delays caspase-dependent cell death and regulates viral spread [79]. Kinases like vacuolar protein sorting 34 (Vps34) complex and cyclin-dependent kinase 1 (Cdk1) are involved in up- or down-regulating autophagy [80].

Further investigation is necessary to understand which cellular mechanisms are targeted by the active compounds identified in this study that result in the disruption of CHIKV-induced CPE and virus dissemination, such as those involved in autophagy or apoptosis. In addition, since CHIKV displays a wide tropism in cell culture. It will be of interest to determine the antiviral activity of these compounds in other relevant cell lines and primary cells that are natural targets of the virus [46], [81]. In summary, the work present here describes the application of a cell-based high-throughput assay system using resazurin and an image-based high content assay approach to screen a kinase-focused chemical library for potential inhibitors of CHIKV. With these two assays, compounds that interfere with CHIKV-induced CPE, which have been shown to play a role in the efficient dissemination of the virus, were identified. The six active compounds identified here could be used to further investigate the mechanisms involved in CHIKV-induced CPE, as well as serve as a starting point for the development of new antiviral drug candidates for CHIKV. Moreover, the cell-based high throughput assay using resazurin and image-based high-content assay systems described here could be applied in the screening other compound libraries for potential CHIKV inhibitors.

## Supporting Information

Figure S1 **Schematic diagram of the CHIKV high-throughput assay using resazurin.** Wells of the 384-well plate are spotted with 10 µM test compounds or 0.5% DMSO vehicle, then incubated with HuH-7 and CHIKV-118-GFP. Cell viability is assessed by measuring converted resorufin at excitation/emission wavelength of 531/572 nm.
(TIF)

Figure S2 **Structural similarity of hit compounds and counter-screening against DENV2.** *Left*: Dendogram showing structural similarity of CHIKV primary hits based on tanimoto similarity index (http://chemmine.ucr.edu). *Right*: percent inhibition of 72 primary hits against CHIKV infection in HuH-7 at 10 µM and percent inhibition/toxicity against DENV2 determined by image-based analysis of DENV2 infection in Huh-7.5. For CHIKV primary hits, shades indicate the following range for percent inhibition: <50% (red), 50%–80% (yellow), >80% (green). For the DENV2 counter-screen, shades indicate the following range: <50% inhibition or >50% toxicity (red), 50%–80% inhibition and <50% toxicity (yellow), >80% inhibition and <50% toxicity (green). Symbols on the right indicate the core scaffold of the 6 CHIKV hit compounds: star (benzofuran), square (thiazol-carboxamide), and diamond (pyrrolopyridine).
(TIF)

Figure S3 **Dose-response curves of 6 confirmed hit compounds.** The inhibition and cytotoxic properties of the 6 CHIKV hit compounds were determined by resazurin reduction assay. HuH-7 was infected with CHIKV-118-GFP (M.O.I. 0.5) for 72 hrs in the presence of the hit compounds at various concentrations. The curves for percent inhibition against CHIKV-118-GFP infection (blue) and cell toxicity (green) are the calculated percent viability based on measured RFU.
(TIF)

Figure S4 **Kinetics of CHIKV-118-GFP infection in HuH-7 measured by GFP expression.** Spread of CHIKV-118-GFP infection in HuH-7 cells at an M.O.I. of 0.5 between 18 and 24 hpi (A). Comparison of infection rate of CHIKV-118-GFP in HuH-7 cells at 3 different multiplicities of infection (0.1, 0.5 and 1.0) between 18 and 24 hpi measured using the in-house image-mining software IM 3.0 (B).
(TIF)

Table S1 **Effect of CHIKV hit compounds on resazurin reduction over 12 hours.**
(DOC)
