# Supplementary figures and images for: Identification of Novel Compounds Inhibiting Chikungunya Virus-Induced Cell Death by High Throughput Screening of a Kinase Inhibitor Library
Source: PLoS Negl Trop Dis. 2013 Oct 31;7(10):e2471. doi: 10.1371/journal.pntd.0002471 (PMC3814572; doi:10.1371/journal.pntd.0002471)

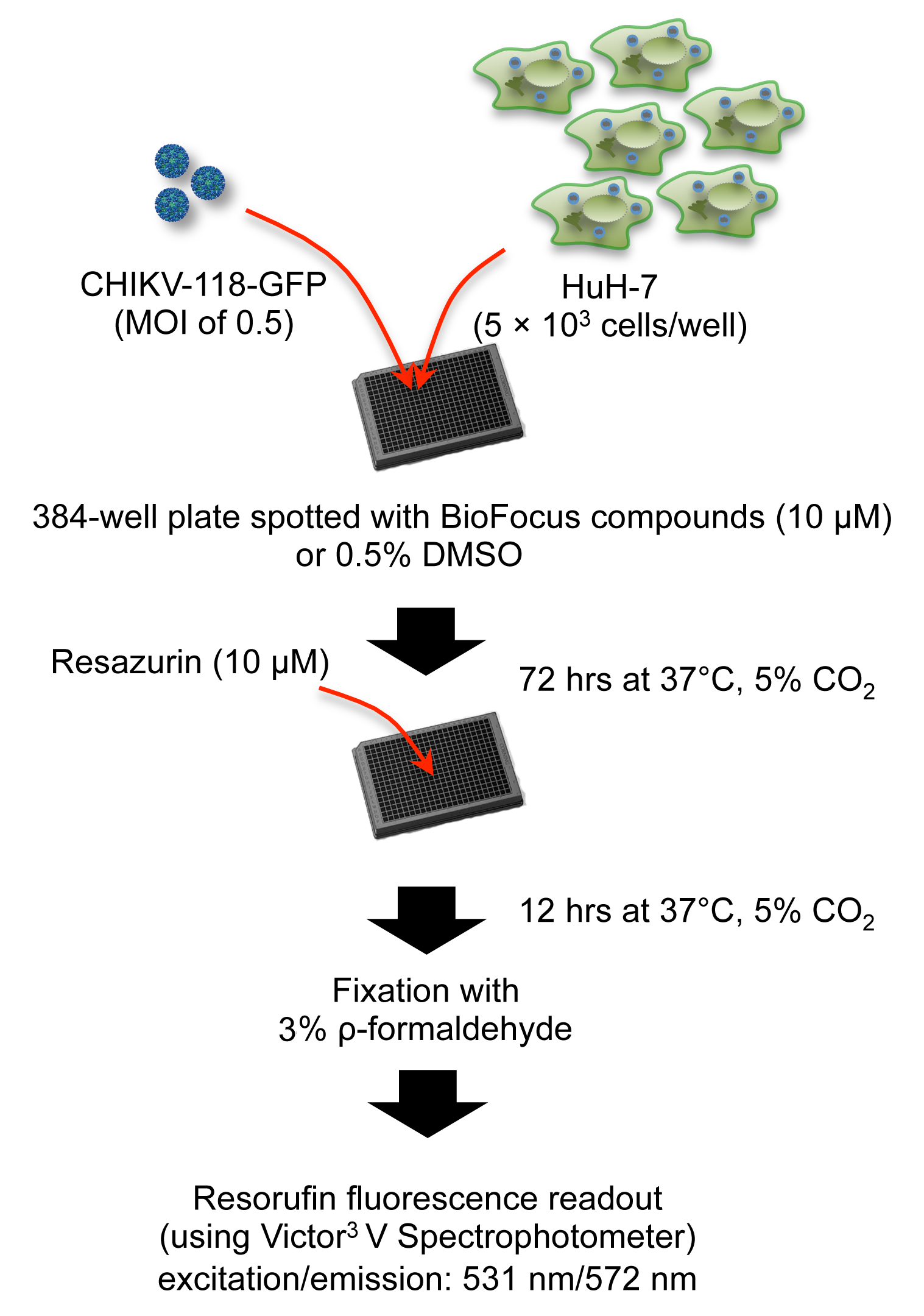

Supplement: Figure S1 — Schematic diagram of the CHIKV high-throughput assay using resazurin. Wells of the 384-well plate are spotted with 10 µM test compounds or 0.5% DMSO vehicle, then incubated with HuH-7 and CHIKV-118-GFP. Cell viability is assessed by measuring converted resorufin at excitation/emission wavelength of 531/572 nm. (TIF) [file pntd.0002471.s001.tif]

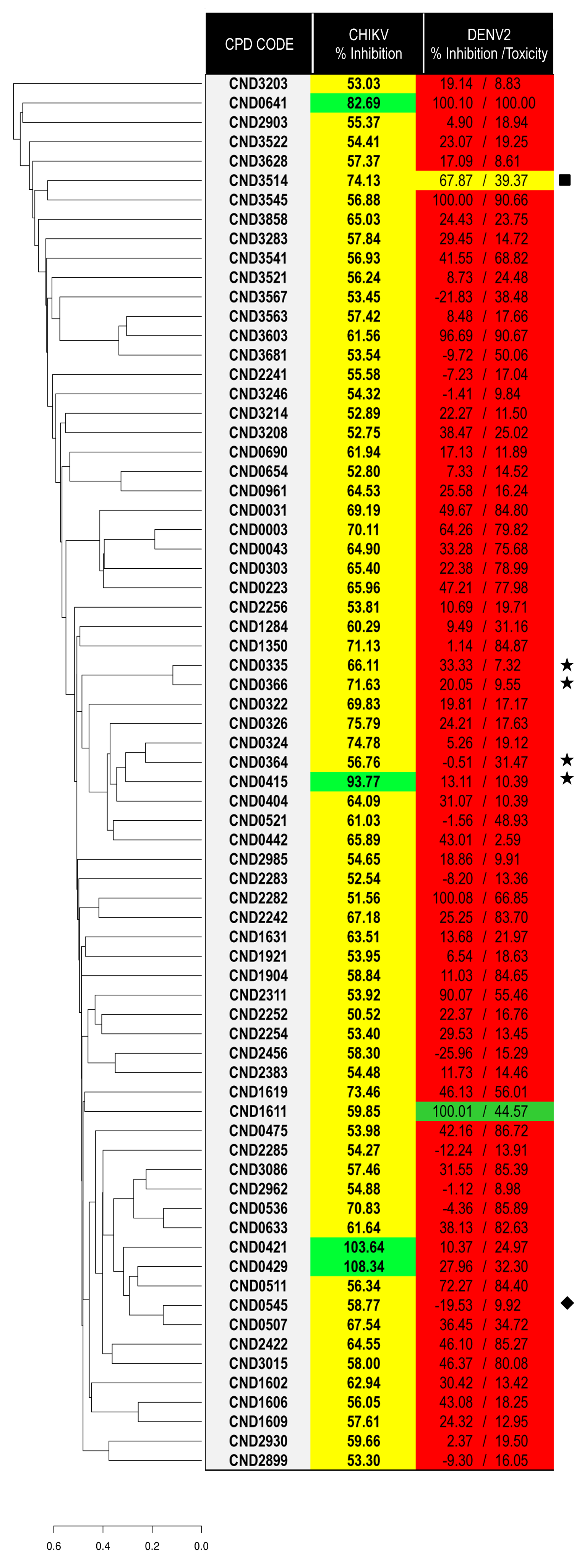

Supplement: Figure S2 — Structural similarity of hit compounds and counter-screening against DENV2. Left: Dendogram showing structural similarity of CHIKV primary hits based on tanimoto similarity index (http://chemmine.ucr.edu). Right: percent inhibition of 72 primary hits against CHIKV infection in HuH-7 at 10 µM and percent inhibition/toxicity against DENV2 determined by image-based analysis of DENV2 infection in Huh-7.5. For CHIKV primary hits, shades indicate the following range for percent inhibition: <50% (red), 50%–80% (yellow), >80% (green). For the DENV2 counter-screen, shades indicate the following range: <50% inhibition or >50% toxicity (red), 50%–80% inhibition and <50% toxicity (yellow), >80% inhibition and <50% toxicity (green). Symbols on the right indicate the core scaffold of the 6 CHIKV hit compounds: star (benzofuran), square (thiazol-carboxamide), and diamond (pyrrolopyridine). (TIF) [file pntd.0002471.s002.tif]

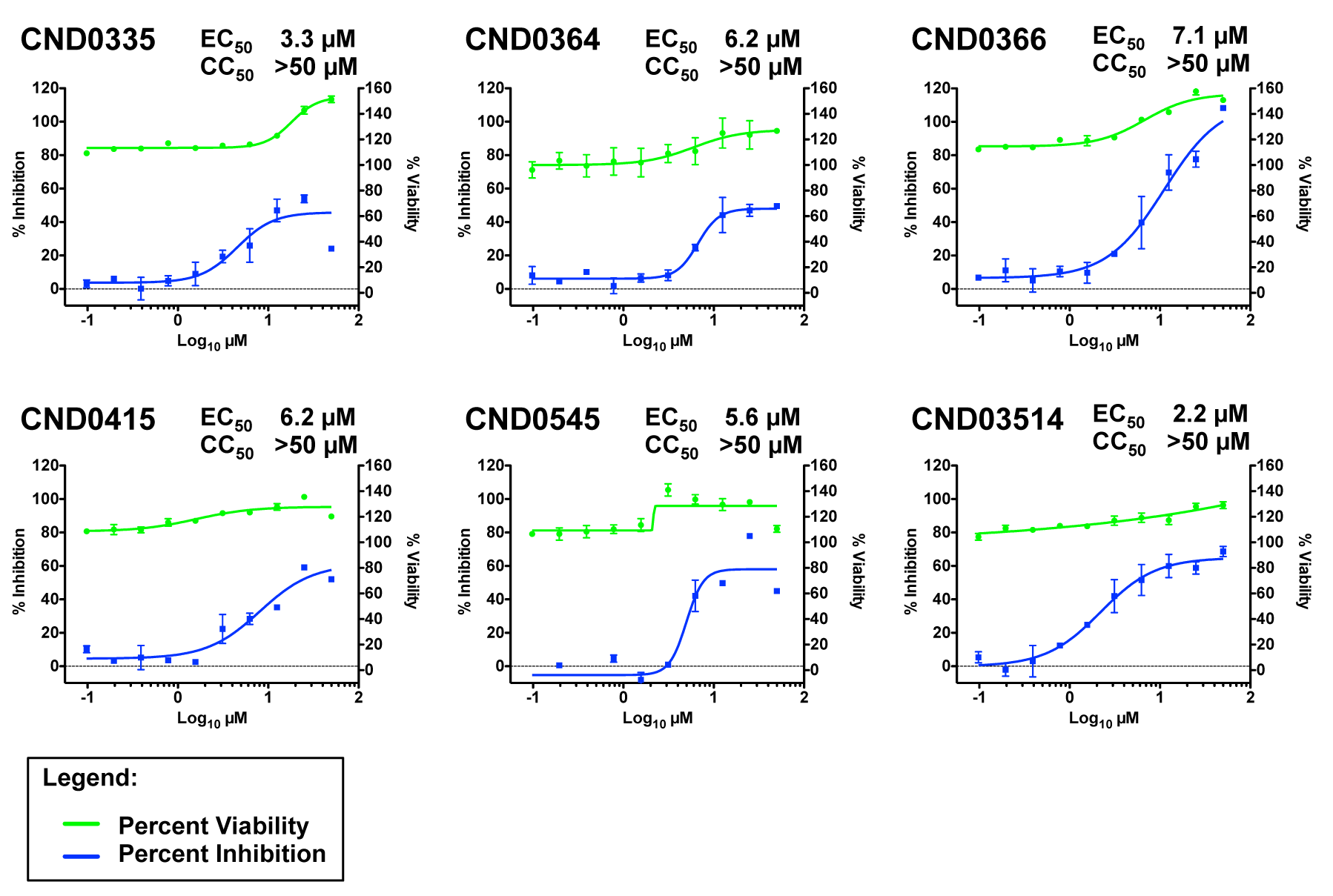

Supplement: Figure S3 — Dose-response curves of 6 confirmed hit compounds. The inhibition and cytotoxic properties of the 6 CHIKV hit compounds were determined by resazurin reduction assay. HuH-7 was infected with CHIKV-118-GFP (M.O.I. 0.5) for 72 hrs in the presence of the hit compounds at various concentrations. The curves for percent inhibition against CHIKV-118-GFP infection (blue) and cell toxicity (green) are the calculated percent viability based on measured RFU. (TIF) [file pntd.0002471.s003.tif]

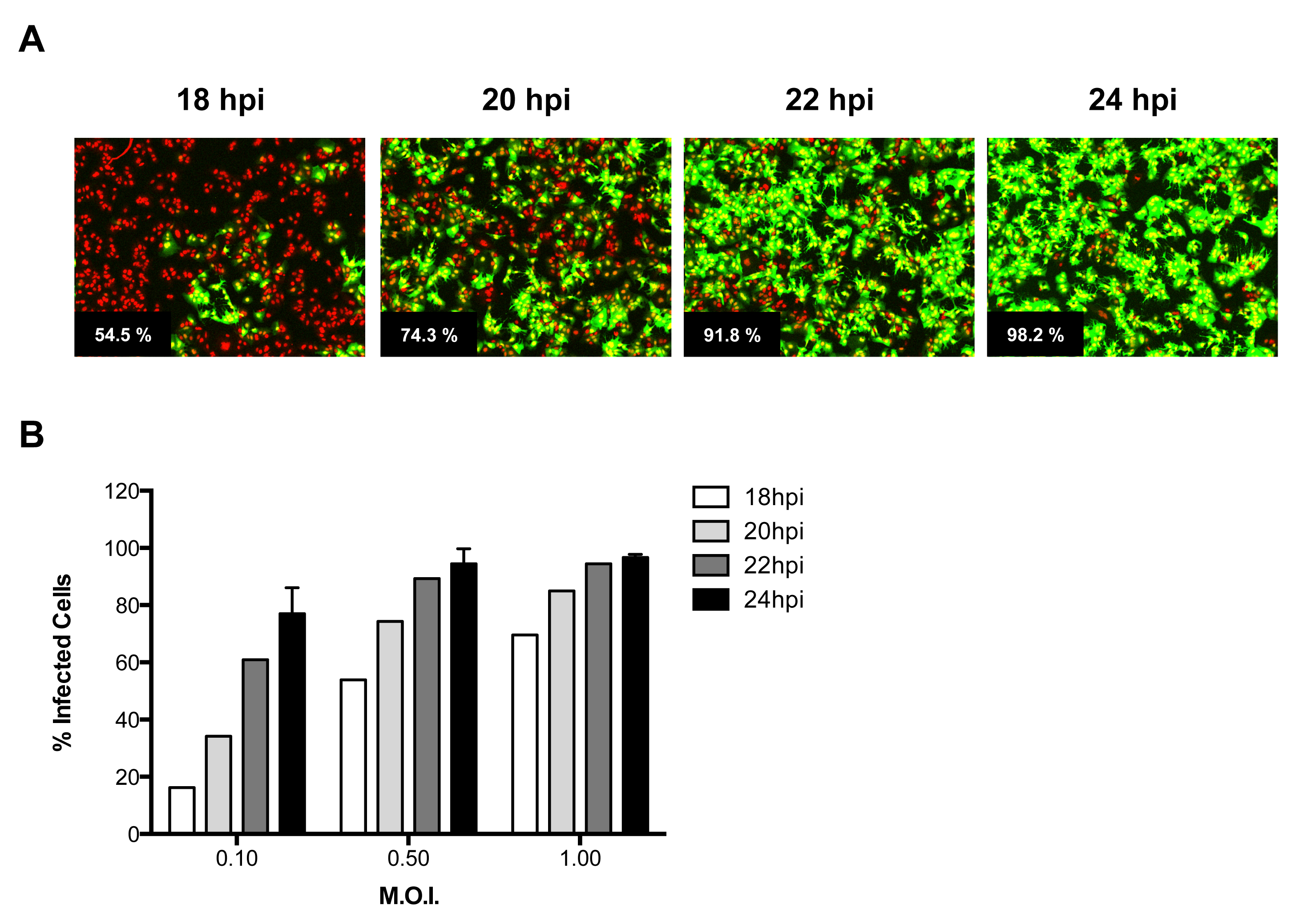

Supplement: Figure S4 — Kinetics of CHIKV-118-GFP infection in HuH-7 measured by GFP expression. Spread of CHIKV-118-GFP infection in HuH-7 cells at an M.O.I. of 0.5 between 18 and 24 hpi (A). Comparison of infection rate of CHIKV-118-GFP in HuH-7 cells at 3 different multiplicities of infection (0.1, 0.5 and 1.0) between 18 and 24 hpi measured using the in-house image-mining software IM 3.0 (B). (TIF) [file pntd.0002471.s004.tif]
