# Supplementary material for: Identification of Novel Compounds Inhibiting Chikungunya Virus-Induced Cell Death by High Throughput Screening of a Kinase Inhibitor Library
Source: PLoS Negl Trop Dis. 2013 Oct 31;7(10):e2471. doi: 10.1371/journal.pntd.0002471 (PMC3814572; doi:10.1371/journal.pntd.0002471)
Supplement: Table S1 — Effect of CHIKV hit compounds on resazurin reduction over 12 hours. (DOC) [file pntd.0002471.s005.doc]

**Table S1.** Effect of CHIKV hit compounds on resazurin reduction over 12 hours.

| **Compound** | **Concentration (µM)** | **RFU (Mean ± SEM)** |
| --- | --- | --- |
| EMPTY |  | 134251 ± 4167 |
| CND0335 | 50.0 | 134468 ± 2782 |
|  | 25.0 | 134083 ± 2617 |
|  | 12.5 | 132612 ± 1892 |
|  | 6.3 | 131901 ± 2890 |
|  | 3.1 | 132843 ± 3615 |
|  | 1.5 | 132660 ± 2366 |
| CND0364 | 50.0 | 127260 ± 1785 |
|  | 25.0 | 126875 ± 548 |
|  | 12.5 | 146299 ± 19909 |
|  | 6.3 | 125566 ± 922 |
|  | 3.1 | 125687 ± 1255 |
|  | 1.5 | 126001 ± 1098 |
| CND0366 | 50.0 | 128162 ± 70 |
|  | 25.0 | 127193 ± 177 |
|  | 12.5 | 126522 ± 346 |
|  | 6.3 | 126123 ± 307 |
|  | 3.1 | 124434 ± 115 |
|  | 1.5 | 124331 ± 405 |
| CND0415 | 50.0 | 123928 ± 552 |
|  | 25.0 | 124969 ± 805 |
|  | 12.5 | 124241 ± 566 |
|  | 6.3 | 124545 ± 453 |
|  | 3.1 | 124287 ± 99 |
|  | 1.5 | 123414 ± 429 |
| CND0545 | 50.0 | 123847 ± 427 |
|  | 25.0 | 124325 ± 663 |
|  | 12.5 | 124622 ± 203 |
|  | 6.3 | 124272 ± 340 |
|  | 3.1 | 124100 ± 341 |
|  | 1.5 | 123282 ± 359 |
| CND3514 | 50.0 | 123902 ± 509 |
|  | 25.0 | 124121 ± 800 |
|  | 12.5 | 124101 ± 904 |
|  | 6.3 | 122856 ± 122 |
|  | 3.1 | 123322 ± 336 |
|  | 1.5 | 123639 ± 696 |

RFU – relative fluorescence unit measured at excitation/emission wavelengths of 531nm/572nm
